# Genome-wide prediction of splice-modifying SNPs in human genes using a new analysis pipeline called AASsites

**DOI:** 10.1186/1471-2105-12-S4-S2

**Published:** 2011-07-05

**Authors:** Kirsten Faber, Karl-Heinz Glatting, Phillip J Mueller, Angela Risch, Agnes Hotz-Wagenblatt

**Affiliations:** 1 Bioinformatics (HUSAR), Core Facility Genomics and Proteomics, German Cancer Research Center, D-69120 Heidelberg, Germany; 2 Division of Epigenomics and Cancer Risk Factors, German Cancer Research Center, D-69120 Heidelberg, Germany

## Abstract

**Background:**

Some single nucleotide polymorphisms (SNPs) are known to modify the risk of developing certain diseases or the reaction to drugs. Due to next generation sequencing methods the number of known human SNPs has grown. Not all SNPs lead to a modified protein, which may be the origin of a disease. Therefore, the recognition of functional SNPs is needed. Because most SNP annotation tools look for SNPs which lead to an amino acid exchange or a premature stop, we designed a new tool called AASsites which searches for SNPs which modify splicing.

**Results:**

AASsites uses several gene prediction programs and open reading frame prediction to compare the wild type (wt) and the variant gene sequence. The results of the comparison are combined by a handmade rule system to classify a change in splicing as “likely, probable, unlikely”. Having received good results from tests with SNPs known for changing the splicing pattern we checked 80,000 SNPs from the human genome which are located near splice sites for their ability to change the splicing pattern of the gene and hereby result in a different protein. We identified 301 “likely” and 985 “probable” classified SNPs with such characteristics. Within this set 33 SNPs are described in the ssSNP Target database to cause modified splicing.

**Conclusions:**

With AASsites single SNPs can be checked for those causing splice modifications. Screening 80,000 known human SNPs we detected about 1,200 SNPs which probably modify splicing. AASsites is available at http://genius.embnet.dkfz-heidelberg.de/menu/biounit/open-husar using any web browser.

## Background

Approximately 6.5 million SNPs have been identified in human genes and have been deposited in the dbSNP database (http://www.ncbi.nlm.nih.gov/projects/SNP/) and are used by the EnsEMBL database (http://www.ensembl.org/). SNP does not only mean exchange of a nucleotide but also a deletion or insertion of one base in the dbSNP database (indels). For many SNPs located in genes the effects on the genes are not known. Application of the new sequencing technologies 454 and Solexa will allow the discovery of many more SNPs which need elucidation of their effects. It is important to know the effect as SNPs can be relevant for diseases e.g. a SNP in the APOE gene increases the risk for developing Alzheimer disease [1]. SNPs account for differences in cancer risk (Dong et al., 2008; Chen et al., 2009) and drug metabolism [2]. Available prediction tools for SNPs like LS-SNP [3] mostly evaluate if the SNP is within a coding region and changes or abolishes the protein. Others contain a collection of previously evaluated SNPs which can be queried by SNP id, disease or chromosomal region [4,5](http://compbio.cs.queensu.ca/F-SNP/). Those SNPs are analysed and scored according to location of the SNP (splice site, ESE, TFBS, coding region) and known effects in diseases. A further list with more than ten web servers which analyze SNPs can be found in Karchin, 2009[6]. In contrast, our tool AASsites looks at the potential of the SNPs to modify the splicing pattern of a gene and does not depend on the annotation of known SNPs. Modified splicing is likely to have a profound effect on the phenotype with relevance to disease risk or drug metabolism. A change in splicing can be caused by modifying any of the components of the splicing machinery such as splice sites or splice enhancers or silencers. Those are evaluated separately to predict a score for modulated splicing by “Skippy” [7]. A new tool called SpliceScanII [8] is looking at all those elements for predicting splice changes in genetic variants and has proven to work in the context of disease-linked variations. AASsites uses the power of gene prediction programs which are trained to evaluate the splice relevant components in order to predict changes in splicing patterns caused by SNPs. Additionally, ESEdetector [9] for discovering changes in ESEs, and programs to detect changes in the open reading frame (ORF) are used. A handmade rule system combines the results and classifies the SNP as “likely”, “probably” or “unlikely” to lead to modified splicing of the gene.

## Results

### The analysis tool AASsites

**Figure 1:**
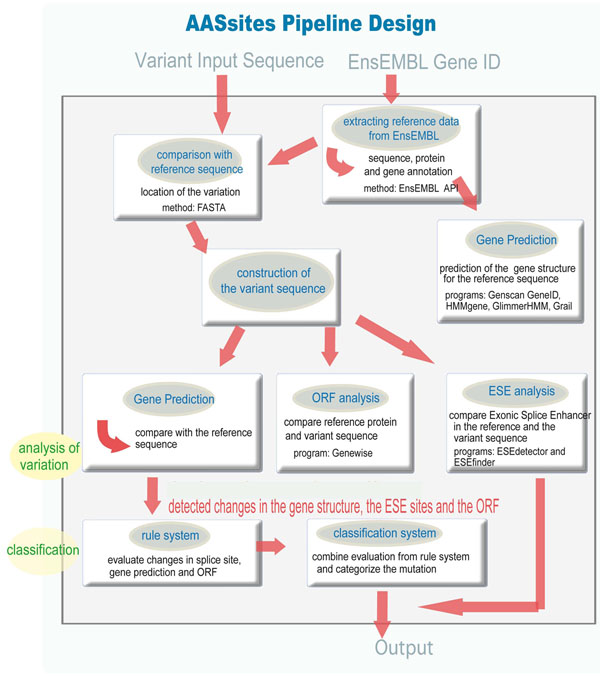
**Overview of the dataflow of the pipeline AASsites**. The different analysis steps performed with the SNP containing input sequence are displayed.

**Figure 2:**
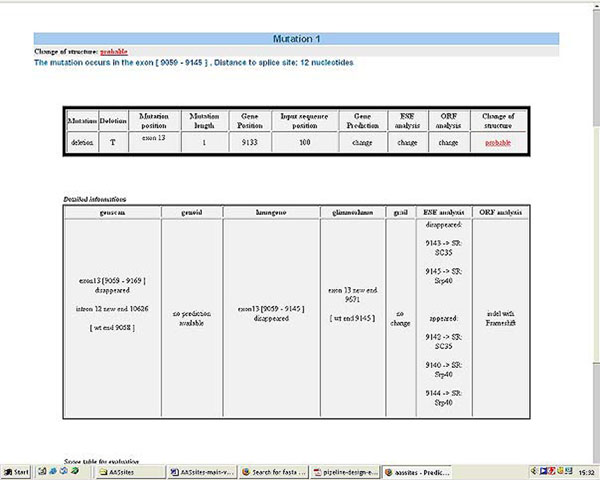
**Example output of pipeline AASsites**. The part of the output with the final classification, the gene predictions and the ORF analysis is shown. The part with the enhancer analysis and the scoring information is omitted.

The tool was designed to analyse one SNP provided within the context of a DNA sequence together with the EnsEMBL gene id (Ensembl53) of the SNP origin. If the input sequence contains more than one SNP belonging to one gene, the different SNPs will be analysed separately. AASsites uses those gene prediction programs capable of correctly predicting the wt intron/exon structure to compare the intron/exon structure of the wt sequence with that of the sequence containing the SNP (see figure 1). Additionally, a change in ESEs and changes in the ORF or amino acid content are checked and reported. Based on the distance of the SNP to the splice site, the predicted changes in the intron/exon structure and the result of the ORF analysis a classification of the SNP into 3 classes is given : likely, probable and unlikely. In the output, details about the gene predictions, ESE changes, ORF and amino acid changes are also given (see figure 2). The tool is available at http://genius.embnet.dkfz-heidelberg.de/menu/biounit/open-husar. It has an average runtime of approximately 3 minutes.

### Test of AASsites using known SNPs

We tested the pipeline AASsites with known example SNPs to evaluate the performance. The database DBASS (database for aberrant splicing, first release, http://www.dbass.org.uk) contains mutations and their experimentally revealed effects on splicing. As described in Methods, 37 SNPs with manually checked and exactly described effects on splicing (positive set 1) and 19 randomly selected mutations from DBASS3 (positive set 2), 23 SNPs causing only an amino acid exchange, and 30 randomly selected SNPs from dbSNP were used for testing AASsites. 66% of the positive SNPs were classified as likely or probable to cause a change in splicing, whereas 100% of all the negative SNPs were classified as unlikely (see Table 1). Overall, 83% of all cases were classified correctly. Looking at the positive set 1 with 37 sequences, in 43% of the cases not only the change in splicing, but also the documented exon/intron modification was predicted correctly. In the negative set, 79% of the cases were predicted as documented. Because of the lack of SNP data with experimentally proven splice changes at that time, the dataset was quite small and cannot be used to provide significant values for sensitivity and specificity. But the test showed that AASsites appears to have a reasonable prediction rate. The comparison with SplicescanII checking for modified or additional/skipped exons showed a better classification rate for AASsites, mainly because it is better in the classification of negative examples.

**Table 1:** Test results of AASsites using SNPs with known changes

Set	Number of SNPs	Correct Classification	Wrong Classification	SpliceScan Correct	SpliceScan Wrong
**Positive**	56	37 (66%)	19 (34%)	32 (57%)	24 (43%)
Negative	53	53 (100%)	0 (0%)	45 (85%)	8 (15%)
All	109	90 (83%)	19 (17%)	77 (71%)	32 (29%)

### Genome-wide analysis of SNPs near splice sites

Since some SNPs are known to be linked with diseases like cancer and play a role in metastasis and resistance of the tumours to drugs, we wanted to screen human genes for SNPs capable of causing changes in splicing of genes. In such a set there should be candidates which can cause a disease by creating a modified protein. Due to the high number of all SNPs which would take up too much computing time we had to reduce the number of SNPs to screen. Because SNPs with low frequencies in the population are not relevant for common diseases we selected SNPs that have a prevalence of over 10% in the population. To raise the chance for identification of splice modifying SNPs we limited the distance to the splice site. Therefore, we selected 82,838 SNPs near splice sites (only intronic SNPs located within 10 bases of the exon-intron boundary and exonic SNPs within 100 bases were considered) with a population frequency above 0.1 in CEU. Those SNPs were run through our AASsites pipeline and the XML output files were parsed via a Perl script. The whole run took about 5 weeks on our linux server (8processors, 16GB RAM). 79,913 SNPs (96% of the selected SNPs) could be analysed by AASsites. 89% of the analysed SNPs were located in an exon, 11 % were located in an intron. The results of the classification are shown in Table 2. 2925 SNPs (4%) could not be analysed, either because the gene was longer than 350kb or because no gene prediction was available.

**Table 2:** Classification results of selected human SNPs

Location	Likely	Probable	Unlikely
Exon	72	430	70444
Intron	239	555	8173
All	311	985	78617

### Identification of SNPs with known splice changes

We compared the SNPs classified as likely or probable to impact the gene’s splicing pattern with SNPs found in the ssSNP target database [10]. In this database SNPs at splice sites with known changes affecting splicing and of relevance for diseases are listed. 33 SNPs identified by AASsites are listed in the ssSNP target database with annotated and experimentally proven changes in splicing and the associated diseases in OMIM, GAD or HGMD (see Additional file 1, Table 2). In 8 cases out of the 33 the splice change predicted by one of the gene prediction programs is exactly the one annotated in the ssSNP entry (see Table 3). The associated diseases are lung cancer [11], renal cell carcinoma [12], tuberous sclerosis [13], hyperglyciaemia [14], prostate cancer [15] and cutis laxa [16] (see Table 3).

**Table 3:** SNPs with known changes in splicing identified by AASsites

Protein	SNP	Change in splice pattern	Associated disease	Reference
GSTM4	rs41283498	Exon skipping	Lung cancer	[11]
PCTK3	rs55957903	Exon skipping	-	
VHL	rs5030815	Exon skipping	Renal cell carcinoma	[12]
TSC2	rs45517091	Exon skipping	Tuberous sclerosis	[13]
GCSH	rs62054483	Exon skipping	Hyperglycinaemia	[14]
NCAN	rs61222528	Exon skipping	-	
EZH2	rs1140478	Exon extension	Prostate cancer	[15]
ATP6V0A2	rs1139788	Exon extension	Cutis laxa	[16]

### Localization of the SNPs modifying splicing

**Figure 3:**
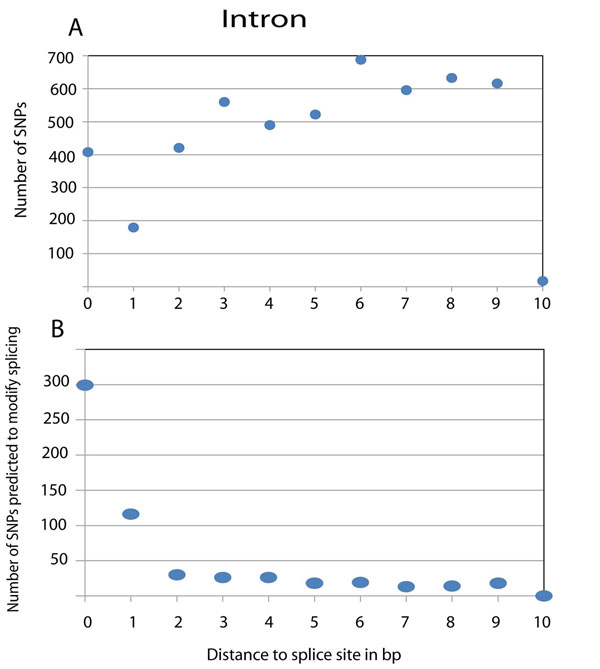
**Distribution of all SNPs and splice modifying SNPs in the intron**. The distribution of all selected SNPs according to the distance to the splice site is shown in panel A, the distribution of splice modifying SNPs in panel B.

**Figure 4:**
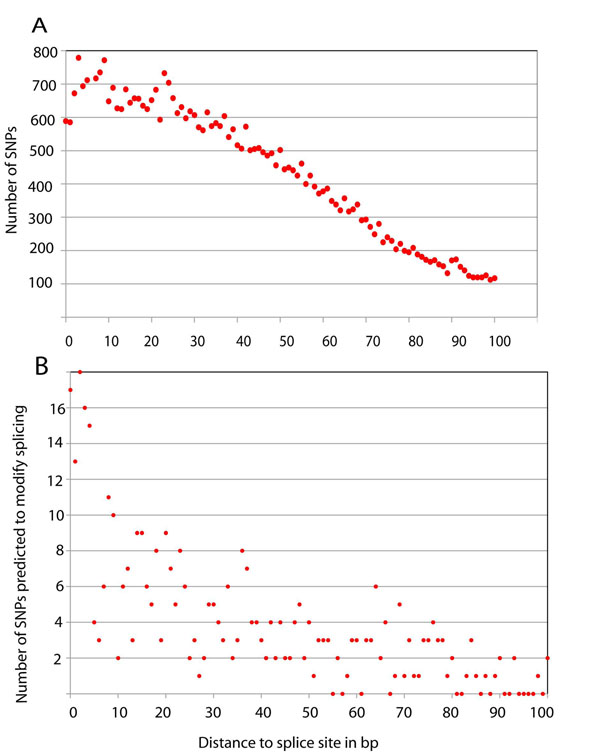
**Distribution of all SNPs and splice modifying SNPs in the exon**. The distribution of all selected SNPs according to the distance to the splice site is shown in panel A, the distribution of splice modifying SNPs in panel B.

The positions of all the SNPs and those of SNPs which were classified as likely or probable are shown in Figure 3 for intronic SNPs and in Figure 4 for exonic SNPs. In the intron (Fig. 3), it is mainly the first two positions starting from the splice site that account for splice variations. In the exon (Fig. 4), the number of splice-modifying SNPs is much lower than in the intron due to the mutation restrictions of the coding sequence. The number of splice relevant SNPs decreases with an increase of the distance to the splice site and shows a steep decline during the first 10 positions in introns and exons, but even at a greater distance some splice modifying SNPs are found. Because of the possible bias of the database towards disease-related gene sequencing, the numbers are likely to be an underestimate.

### Pathway distribution of the genes with SNPs modifying splicing

We have analyzed the genes according to their annotated pathways with the DAVID tools (http://david.abcc.ncifcrf.gov/). Of originally 1300 SNPs on 971 genes, the DAVID database recognized 711 genes according to their EnsEMBL identifiers. The analysis produced a table of 187 genes with their pathways. The over-represented pathways are shown in Table 4. Among the top over-represented pathways ones like “Focal adhesion”, “Metabolism of xenobiotics by cytochrome P450”, and “ABC transporters general” can be found. These pathways are very often disturbed in cancer cells.

**Table 4:** Pathways over-represented in genes with SNPs modifying splicing

Pathway	Count	%	P-Value
Focal adhesion	30	3.5	5.9E-6
ECM-receptor interaction	17	2.0	5.7E-5
Metabolism of xenobiotics by cytochrome P450	12	1.4	2.3E-3
ABC transporters - General	9	1.1	4.9E-3
Bladder cancer	7	0.8	3.1E-2
Regulation of actin cytoskeleton	21	2.5	3.3E-2
Adherens junction	10	1.2	3.3E-2
Phenylpropanoid biosynthesis	3	0.4	6.3E-2
Colorectal cancer	10	1.2	7.0E-2
Small cell lung cancer	10	1.2	7.8E-2
Cyanoamino acid metabolism	3	0.4	8.0E-2
Non-small cell lung cancer	7	0.8	9.2E-2
Pathogenic Escherichia coli infection - EPEC	7	0.8	9.8E-2

The overexpressed pathways are shown together with the number of genes involved in the pathway (Count), the percentage of genes involved in the pathway compared to all input genes (%), and the p value from the Fisher test calculated by the DAVID tool.

## Discussion

To identify SNPs which modify the protein by changing the splicing pattern the pipeline AASsites was developed. This pipeline is available through its web interface at http://genius.embnet.dkfz-heidelberg.de/menu/biounit/open-husar. Unlike many other SNP analysis tools our tool predicts the effect of SNPs on splicing. Not only SNPs localized at splice sites can modify the splicing of a gene, but also SNPs near splice sites can have the same effect due to other regulatory sequences involved. Gene prediction programs take these regulatory sequences into account by using HMM models or similar algorithms. Still, we could not predict 433 genes because in these cases none of the five gene prediction programs worked correctly on the wt sequence. This minor problem could be solved by the implementation of one or two more prediction tools. A second problem is the prediction of SNPs in alternatively spliced products. Most gene prediction programs do not predict alternative splice sites. The only exception is Augustus [17](http://augustus.gobics.de) which should be implemented. Then also the different alternatively spliced wild type forms of the gene have to be considered.

We have shown with a set of SNPs known to affect or not to affect splicing, that the pipeline was able to correctly predict the change in splicing caused by the SNP in 83% of 109 cases. The problem of testing and improving the rule system for combining the results lies in the small number of experimentally proven SNP-derived modifications in splicing. With more experimental data available we could replace the rule system by a knowledge system based on machine learning algorithms or we could optimize the rules. The comparison with SpliceScanII [8] shows that AASsites performs better on our small test set. But the number of examples is much too small for a final evaluation.

New tools could be implemented to assist AASsites by selecting the correct splice change if different changes are predicted by the different gene prediction tools. A further analysis of the predicted splice sites with tools like the “Human Splicing Finder” [18] which predicts the effect of mutations on the splice signals or “Skippy” [7] which analyses ESEs and ESSs and the evolutionary constraint of the region surrounding the variant could complement our approach.

Another improvement could be the evaluation of different SNPs of the same haplotype together. At the moment, AASsites treats all SNPs as being independent. The analysis is done for only one SNP at a time, even if the input sequence contains several SNPs. That is the reason, that the combined effects of multiple SNPs are missed.

The genome-wide analysis of known SNPs near splice sites revealed 1300 SNPs which are probably capable of modifying the protein by changed splicing. It could be shown, that not only SNPs directly at splice sites are likely to modify splicing. Among the splice relevant SNPs were 33 cases which were experimentally verified and involved in the genesis of diseases according to the ssSNP target database proving the functionality of the pipeline. Other SNPs in genes which are related to diseases were found and could be candidates for further research.

## Conclusions

To identify SNPs which modify the protein by changing the splicing pattern the pipeline AASsites was developed. This pipeline uses gene prediction programs for this purpose and is available through its web interface at http://genius.embnet.dkfz-heidelberg.de/menu/biounit/open-husar. The genome-wide analysis of human SNPs near splice sites revealed 1300 SNPs which are probably capable of modifying the protein by changed splicing. Some already known SNPs were identified, but other SNPs in genes related to diseases could be good candidate SNPs for further research.

## Methods

### AASsites Pipeline Design

An overview of the AASsites pipeline is outlined in Figure 1. Input is a DNA sequence containing the SNP and the EnsEMBL gene id (EnsEMBL version 53) to which the SNP belongs. The EnsEMBL gene id is used to extract the wt genomic sequence and the wt protein as well as to derive the real exon-intron structure. The different analysis steps which are outlined below are performed with the SNP containing sequence. An HTML report page with the classification and the single results (see Figure 2) is produced as output.

### Localization of the SNP

The input DNA sequence is compared to the wt sequence by the FASTA program [19]. The position of the SNP determines its location in an intron or an exon. Depending on the location – intron or exon - a different set of tools is run and different rules are applied.

### Gene prediction programs used

At the moment five different gene prediction programs are implemented into the AASsites pipeline. They rely on different models for prediction.

GenScan [20] is based on hidden markov models and considers elementary signals like basic transcriptional, translational and splicing signals as well as length distributions and compositional features of exons, introns and intergenic regions.

Class Hidden Markov models are used in HMMgene [21] to predict the most probable gene structure based directly on labelled sequences, using labels for coding regions, introns and intergenic regions.

The program GeneID [22] uses a hierarchical approach composed of three different steps to assemble the gene structure. It starts out by scoring splice sites, start and stop codon using so-called Position Weight Matrices (PWMs). In the second step, exons are built from the sites. Exons are scored as the sum of the scores of the defining sites, plus the log-likelihood ratio of a Markov Model for coding DNA. In the last step, from the set of predicted exons, the gene structure is assembled, maximizing the sum of the scores of the assembled exons.

A generalised HMM is the basis of GlimmerHMM [23], which also uses decision trees and the maximal dependence decomposition method.

The last program, GrailEXP6 [24](http://grail.lsd.ornl.gov/grailexp/index.html), is implemented as a building block system consisting of three different parts. It first uses statistical techniques to pinpoint possible locations of exons. Then it brings in empirical evidence from nucleotide and protein databases to create possible "pieces" of genes. Finally, an intelligent algorithm constructs the genes from these pieces.

### Getting the information for the wt sequence

To determine possible changes due to the SNP, the wt sequence and information about the structure have to be determined. Using the EnsEMBL Perl API (Ensembl53) the wildtype sequence, the intron-exon structure and the protein sequence are extracted from the EnsEMBL database.

### Selecting the gene prediction programs to be run

Five gene prediction programs are used to predict the gene structure of the wildtype gene sequence. These predictions are compared independently to the gene structure derived from EnsEMBL. A given gene prediction program is used for the prediction of the sequence containing the mutation if the exon or intron, in which the SNP is localised, was correctly predicted for the wildtype sequence. This selection means that not all prediction programs are used for each SNP. If no prediction program can be found to predict the wt exon or intron, the program will output “No prediction available”. The predicted gene structures for the SNP-containing sequence are compared to the wildtype structure to detect changes.

### Analysis of the Open Reading Frame (ORF)

Using the GeneWise program [25] changes in the Open Reading Frame are analysed. GeneWise combines a gene structure model and a homology model to predict the protein sequence for a genomic sequence and to compare this sequence with a homologous protein sequence. In AASsites 100 coding basepairs of the variant sequence around the SNP are analysed with GeneWise.

### Observation of the Exonic Splicing Enhancers (ESEs)

If a SNP is localised in an exon the ESEs are analysed with ESEfinder [26] or ESEdetector [9]. The prediction of putative ESEs in query sequences performed by ESEfinder is based on weight matrices corresponding to the motifs of four different human SR proteins. The values that constitute the matrices are derived from frequency values obtained from the alignment of so called winner sequences of the SELEX experiments. ESEdetector is based on a support vector machine and uses a combined oligo-kernel to predict possible Exonic Splicing Enhancers in an input-sequence. It has a better prediction accuracy than ESEfinder but needs exons >=100bp. AASsites uses ESEdetector to predict ESE elements in the wildtype and in the variant exon of at least 100bp, otherwise it uses ESEfinder. Up to 300bp of the exon are taken into account and ESE elements in wildtype exon and variant exon are compared.

### Scoring and rule system for combining the different predictions

The details of the scoring system are shown in Table 5. Low scores are given to the cases in which splice changes would be expected e.g. by a variation at the splice site, a high score if no change is expected. As shown in Table 5 the distance of the SNP to the splice site, the changes predicted by the gene prediction programs and the ORF changes are scored. Scores 1-4 are given according to the changes predicted by the gene prediction programs evaluating the majority prediction (see Table 5). The distance of the SNP to the splice site is also scored. In the case that the SNP is located in an intron, those scores are combined and determine the final prediction, the rules for which can be seen in Table 6. If the SNP is located in an exon, an additional score (ORF) takes the changes to the ORF by the SNP into account (see Table 5). The scores ORF 1 and ORF 2 are only for sequence variations other than SNPs which only exchange one nucleotide. The different scores are then combined according to the rules given in Table 6. Those rules combine different lower scores to ‘probable’ or ‘likely’, the higher scores to ‘unlikely’. ORF and gene prediction scores decide about the final classification in most cases.

**Table 5:** Scoring table for combining the results of the AASsites analysis tools

Score	1	2	3	4	5	0
SNP distance to splice site	<=2 nt	>2 nt and <=4 nt	>4 nt	-	-	-
Gene prediction	Intron/ Exon disappared/ appeared	Intron/Exon modified	No change	No prediction available	-	-
ORF	Indel with frameshift	Indel without frameshift	No frameshift no stop-codon appeared	New Amino Acid	No genewise prediction	Stop-codon appeared

**Table 6:** Classification rules

SNP location	Rule*	Classification
Exon	ORF 1 or 0	probable
Exon	ORF 2 and Gene prediction 1	likely
Exon	ORF 2 and (Gene prediction 2 or 3 or 4)	unlikely
Exon	ORF 3 or 4	unlikely
Exon	ORF 5 and (Gene prediction 1 or 2) and (SNP distance 1 or 2)	likely
Exon	ORF 5 and ((Gene prediction 3 or 4) or (SNP distance 3 or 4))	unlikely
Intron	SNP distance 1 and (Gene prediction 1 or 2)	probable
Intron	SNP distance 1 and (Gene prediction 3 or 4)	unlikely
Intron	SNP distance 2 and (Gene prediction 1 or 2)	likely
Intron	SNP distance 2 and (Gene prediction 3 or 4)	unlikely
Intron	SNP distance 3 and (Gene prediction 1 or 2)	likely
Intron	SNP distance 3 and (Gene prediction 3 or 4)	unlikely

* Rule means the combination of scores for the different features from table 5

### Test set of SNPs with known changes

The database DBASS (database for aberrant splicing, first release, http://www.dbass.org.uk) contains mutations and their experimentally revealed effects on splicing. Using this database and the referred publications, a set of 37 SNPs could be selected which affected the splice pattern in a defined way (positive set 1). Added to this set was a randomly chosen set of 19 SNPs of DBASS3, not manually checked (positive set 2). As a negative set 1 23 SNPs were chosen which cause an amino acid exchange only. The SNPs of the positive set 1 and negative set 1 together with the described effects and the publications are shown in Additional file 1, Table 1. Additionally 30 SNPs randomly selected from dbSNP were used as a negative set 2, as splice modifying SNPs are rare and should not appear in a small randomly selected set. In this set 17 intronic SNPs are included. SpliceScanII [8] was run on all wt and variant sequences with default parameters and compact output. The differences in exon numbers or exon start/stop sites were counted as a predicted splice modification of the variant.

### Selection of SNPs for the genome wide analysis

Approximately 5 million human SNPs located in protein coding genes and found in EnsEMBL 53 (http://www.ensembl.org) were the starting point. Assuming that SNPs near splice sites are more likely to be involved in splice changes, only intronic SNPs located within 10 bases of the exon-intron boundary and exonic SNPs within 100 bases of the splice site were considered. Additionally, a population frequency of the SNP of at least 0.1 in the CEU population was required. According to the described criteria 82,838 SNPs were selected by a perl script which used the EnsEMBL API for extracting the SNPs, the splice sites, the sequences and the population frequencies.

## Authors' contributions

Designed the program: KF AR PJM KHG AHW, interpreted data: AR PJM, programmed and tested the tool: KF KHG PJM, wrote the manuscript: KF AHW AR, all authors read and approved the manuscript.

## Competing interests

The authors declare that they have no competing interests.

## Supplementary Material

Additional file 1 This file contains two tables. Table 1 lists the SNPs used in set 1 for benchmarking AASsites together with their references. Table 2 displays the SNPs identified by the genome wide analysis of AASsites that are listed in the ssSNP target database together with their associated diseases. Click here for file
