# Supplementary material for: Genome-wide prediction of splice-modifying SNPs in human genes using a new analysis pipeline called AASsites
Source: BMC Bioinformatics. 2011 Jul 5;12(Suppl 4):S2. doi: 10.1186/1471-2105-12-S4-S2 (PMC3194194; doi:10.1186/1471-2105-12-S4-S2)
Supplement: Additional file 1 — This file contains two tables. Table 1 lists the SNPs used in set 1 for benchmarking AASsites together with their references. Table 2 displays the SNPs identified by the genome wide analysis of AASsites that are listed in the ssSNP target database together with their associated diseases. [file 1471-2105-12-S4-S2-S1.pdf]

Additional Material

Table 1: SNPs used for benchmarking AASsites

POSITIVE SET				
Gen CHRNE	AA change	Mutation	Position	Reference
CHRNE_1		g.IVS6-1G>C*	3' splice site of intron 6	Ohno *et al.*, 2005
CHRNE_3		g.IVS9-1G>A*	3'splice site of intron 9	
CHRNE_4		g.IVS10-9_c.1167dup16*	16 bp duplication comprising 8 bp at 3' end of intron 10 and 8bp of 5'end of exon 11	
CHRNE_5		c.1259_g.IVS11+15del23	23 bp deletion comprising 8 bp at 3' end of exon 11 and 15 bp at 5' end of intron 11	
CHRNE_6		c.857G>T (p.R286M)	G->T substitution at 3' end of exon 8	
CHRNE_7		g.IVS4-2A>C	3' splice site of intron 4	
CHRNE_8		g.IVS6+1G>T	5' splice site of intron 6	
CHRNE_9		g.IVS7+2T>C	5' splice site of intron 7	
CHRNE_10		g.IVS10+2T>G	5' splice site of intron 10	
Gen GLA	AA change	Mutation	Position	Reference
GLA_1		g.IVS3-1G>C	3' splice site of intron 3	Germain et al, 1999
Gen MAN2B1	AA change	Mutation	Position	Reference
MAN2B1_1		g.IVS5-1G>C	AG->AC in acceptor site of intron 5	Berg *et al.*, 1999

MAN2B1_2		g.IVS7+2T>G	GT->GG in donor site of intron 7	
MAN2B1_3		g.IVS11-3del25	Deletion of 25 bp preceeding -2 in acceptor site of intron 11	
MAN2B1_4		g.IVS12+2T>G	GT->GG in donor site of intron 12	
MAN2B1_5		g.IVS14+1G>C	GT->CT in donor site of intron 14	
MAN2B1_6		g.IVS14-2A>G	AG->GG in acceptor site of intron 14	
MAN2B1_7		g.IVS17+1G>A	GT->AT in donor site of intron 17	
MAN2B1_8		g.IVS17+1G>T	GT->TT in donor site of intron 17	

Gen PRPF31	AA change	Mutation	Position	Reference
PRPF31_1		g.IVS5-1G>A	at the position 1 of the Intron 5	Xia et al., 2004

Gen HMBS	AA change	Mutation	Position	Reference
HMBS_3		c.900delT	Deletion of a single T in exon 14	De Siervi et al., 1999

Gen ATM	AA change	Mutation	Position	Reference
ATM_0		g.IVS29-1G->A	At position 1 of intron 29	Eng *et al.*, 2004
ATM_2		g.IVS20-597delAAGT	At position 579 of intron 20	
ATM_3	W2483X	c.7449G->A	At position 7449 (exon 52)	
ATM_4	A2622V	c.7865C->T	At position 7865 (exon 55)	
ATM_5	Y171Y	c.513C->T	At position 513 (exon 8)	
ATM_6		g.IVS11-2A->G	At position 2 of intron 11	
ATM_7		g.IVS37-5delTCTA	At position 5 of intron 37	
ATM_8		g.IVS21-2A->G	At position 2 of intron 21	
ATM_9		g.IVS38-2A->C	At position 2 of intron 38	

Gen OPA1	AA change	Mutation	Position	Reference
OPA1_1		c.2014-1G>A	Intron 20	Schimpf et al., 2006

OPA1_2		c.2013G>A	Exon 20	
OPA1_3		c.1705+1G>T	Intron 17	
OPA1_4		c.2708-1G>T	Intron 26	
OPA1_5		c.1141G>A	Exon 11	
OPA1_7		c.2354A>G	Exon 23	
OPA1_8		c.2614-9A>G	Intron 25	

NEGATIVE SET

Gen ADA	AA change	Mutation	Position	Reference
ADA_1	V177M	c.529G>A	Exon 6(bp 529)	Santisteban *et al.*, 1993
ADA_2	R101L	c.302G>T	Exon 4 (bp 302)	
ADA_3	S291L	c.872C>T	Exon 10 (bp 872)	
ADA_4	R156H	c.467G>A	Exon 5 (bp 467)	
ADA_5	G216R	c.646G>A	Exon 7 (bp 646)	

Gen GLA	AA change	Mutation	Position	Reference
GLA_10	C202W	TGT->TGG	Exon 4 (cdna bp 606)	Germain et al., 1999
GLA_11	C223G	TGC->GGC	Exon 5 (cdna bp 667)	
GLA_12	N224D	AAT->GAT	Exon 5 (cdna bp 670)	
GLA_13	R301Q	CGA->CAA	Exon 6 (cdna bp 902)	
GLA_14	Q327K	CAA->AAA	Exon 6 (cdna bp 979)	

Gen HMBS	AA change	Mutation	Position	Reference
HMBS_1	R149Q	CGA->CAA	Exon 9 (bp 446)	De Siervi et al.,

				1999
HMBS_2	L245L	CTT->CTG	Exon 12 (bp 735)	

Gen MAN2B1	AA change	Mutation	Position	Reference
MAN2B1_9	H72L	A->T	Exon 2 (bp 215)	Berg et al., 1999
MAN2B1_10	T355P	A->C	Exon 8(bp 1063)	
MAN2B1_11	P356R	C->G	Exon 8 (bp 1067)	
MAN2B1_12	E402K	G->A	Exon 9 (bp 1204)	
MAN2B1_13	W714R	T->C	Exon 17 (bp 2140)	
MAN2B1_14	R750W	C->T	Exon 18 (bp 2248)	
MAN2B1_15	L809P	T->C	Exon 20 (bp 2426)	

Gen PKP2	AA change	Mutation	Position	Reference
PKP2_1	S140F	C->T	Exon 3 (bp 419)	Gerull *et al.*, 2004
PKP2_2	S615F	C->T	Exon 9 (bp 1844)	
PKP2_3	K654Q	A->C	Exon 9 (bp 1960)	
PKP2_4	C796R	T->C	Exon 12 (bp 2386)	

References for Table 1 :

Berg, T., Riise, H. M., Hansen, G. M., Malm, D., Tranebjaerg, L., Tollersrud, O. K., & Nilssen, O. 1999. Spectrum of mutations in alpha-mannosidosis. *Am J Hum Genet*, **64**(1), 77–88.

De Siervi, A., Mendez, M., Parera, V.E., Varela, L., Batlle, A.M. and Rossetti, M.V. (1999) Acute intermittent porphyria: characterization of two novel mutations in the porphobilinogen

deaminase gene, one amino acid deletion (453-455delAGC) and one splicing aceptor site mutation (IVS8-1G>T). Hum Mutat, 14, 355

Eng, Laura, Coutinho, Gabriela, Nahas, Shareef, Yeo, Gene, Tanouye, Robert, Babaei, Mahnoush, D¨ork, Thilo, Burge, Christopher, & Gatti, Richard A. 2004. Nonclassical splicing mutations in the coding and noncoding regions of the ATM Gene: maximum entropy estimates of splice junction strengths. *Hum Mutat*, **23**(1), 67–76.

Germain, D. P., & Poenaru, L. 1999. Fabry disease: identification of novel alpha-galactosidase A mutations and molecular carrier detection by use of fluorescent chemical cleavage of mismatches. Biochem Biophys Res Commun, 257(3), 708–713

Gerull, Brenda, Heuser, Arnd, Wichter, Thomas, Paul, Matthias, Basson, Craig T, McDermott, Deborah A, Lerman, Bruce B, Markowitz, Steve M, Ellinor, Patrick T, MacRae, Calum A, Peters, Stefan, Grossmann, Katja S, Drenckhahn, J¨org, Michely, Beate, Sasse-Klaassen, Sabine, Birchmeier, Walter, Dietz, Rainer, Breithardt, G¨unter, Schulze-Bahr, Eric, & Thierfelder, Ludwig. 2004. Mutations in the desmosomal protein plakophilin-2 are common in arrhythmogenic right ventricular cardiomyopathy. *Nat Genet*, **36**(11), 1162–1164.

Ohno, K., Tsujino, A., Shen, X-M., Milone, M., & Engel, A. G. 2005. Spectrum of splicing errors caused by CHRNE mutations affecting introns and intron/exon boundaries. *J Med Genet*, **42**(8), e53.

Santisteban, I., Arredondo-Vega, F. X., Kelly, S., Mary, A., Fischer, A., Hummell, D. S., Lawton, A., Sorensen, R. U., Stiehm, E. R., & Uribe, L. 1993. Novel splicing, missense, and deletion mutations in seven adenosine deaminase-deficient patients

with late/delayed onset of combined immunodeficiency disease. Contribution of genotype to phenotype. *J Clin Invest*, **92**(5), 2291–2302.

Schimpf, Simone, Schaich, Simone, & Wissinger, Bernd. 2006. Activation of cryptic splice sites is a frequent splicing defect mechanism caused by mutations in exon and intron sequences of the OPA1 gene. *Hum Genet*, **118**(6), 767–771.

Xia et al. (2004) A novel PRPF31 splice-site mutation in a Chinese family with autosomal dominant retinitis pigmentosa. Mol. Vis., 10

Table 2: List of SNPs identified by AASsites and contained in the ssSNPTarget database*

Chr.	SNP	Described Change	Gene name (ENSembl ID)	Affected domain	Disease
1	rs61811105		AGL (ENSG00000162688)		Glykogenase
1	rs55888274		CR1 (ENSG00000203710)		Tuberculosis; Lung fibrosis;
1	rs4690223		IDUA (ENSG00000127415)		Hurler Syndrom
1	rs41313932		HAX1 (ENSG00000143575)		Neutropenia
1	rs41283498	Exon Skipping	GSTM4 (ENSG00000168765)		Lung cancer
1	rs41283130		HSD11B1 (ENSG00000117594)	ADH Zink-bindenene Dehydrogenase	polycystic ovarial syndrome; High blood pressure; Diabetes Type II;

					Alzheimer disease
1	rs61726477		LMNA (ENSG00000160789)	Filamentprotein	Ertherosclerosis
1	rs55957903	Exon Skipping	PCTK3 (ENSG00000117266)		
3	rs5030815	Exon Skipping	VHL (ENSG00000134086)	HVL (Hippel-Landau tumor suppression protein	Kidney carcinoma
4	rs55737343		KIT (ENSG00000157404)		Leukemia
5	rs60770849		ITGA1 (ENSG00000213949)		Diabetes Type II; liver disease
6	rs1126474		GLP1R (ENSG00000112164)	7 transmembrane receptor	Diabetes Type II
7	rs1140478		EZH2 (ENSG00000106462)		Prostate cancer
10	rs57979136		SLC18A2 (ENSG00000165646)		Parkinson disease
11	rs58174038		SLC22A12 (ENSG00000197891)	Transporter of sugar	Kidney disease; Hyperurikemia
12	rs1139788	Exon Extension	ATP6V0A2 (ENSG00000185344)	V-Typ ATPase 116kDA family	Cutix Laxa
15	rs55966303		LRRK1 (ENSG00000154237)	Domain of the RAS family	Parkinson disease
16	rs36023846		SCNN1B (ENSG00000168447)		High blood pressure
16	rs4548893		AC093520.4 (ENSG00000214645)		Tuberculosis
16	rs45517091	Exon Skipping	TSC2 (ENSG00000103197)		Tuberculosis

16	rs57263696		CES2 (ENSG00000172831)	Alpha-Beta Hydrolase; Prolyl-Oligopeptidase Family	Cancer
16	rs62054483	Exon Skipping	GCSH (ENSG00000140905)	Glycin spaltendes H-Protein	Hyperglycinamia
18	rs56302501		GNAL (ENSG00000141404)	Domain of the G-Protein Alpha subunit	Dbipolare Störungen
19	rs36000627		ACTN4 (ENSG00000130402)	Calponin Homolog	Glomerulosclerosis
19	rs1833783		FTL (ENSG00000087086)	Ferritin similar	Parkinson disease
19	rs34044708		GAPDHS (ENSG00000105679)	Glyceraldehyd Domain	Alzheimer disease
19	rs61222528	Exon Skipping	NCAN (ENSG00000130287)	Lectine C-Typ Domain	
19	rs59309543		PRKCG (ENSG00000126583)	Tyrosine kinase Domain	Spinocerebellar ataxia
20	rs11547860		SNAP25 (ENSG00000132639)		Attention-deficit hyperactivity disorder
22	rs2330844		GGT1 (ENSG00000100031)		Glutathiononuria
22	rs5027908		SHANK3 (ENSG00000099882)	SH3 Domain	Autism
X	rs10465407		SLC25A14 (ENSG00000102078)	Mitochondrial transport protein	Schizophrenia

*) ssSNPTarget is a database of genome wide splice-site SNPs
